# Pharmacovariome scanning using whole pharmacogene resequencing coupled with deep computational analysis and machine learning for clinical pharmacogenomics

**DOI:** 10.1186/s40246-023-00508-1

**Published:** 2023-07-14

**Authors:** Alireza Tafazoli, John Mikros, Faeze Khaghani, Maliheh Alimardani, Mahboobeh Rafigh, Mahboobeh Hemmati, Stavroula Siamoglou, Agnieszka Kitlas Golińska, Karol A. Kamiński, Magdalena Niemira, Wojciech Miltyk, George P. Patrinos

**Affiliations:** 1 Department of Analysis and Bioanalysis of Medicines, Faculty of Pharmacy With the Division of Laboratory Medicine, Medical University of Bialystok, 15-089 Białystok, Poland; 2 Laboratory of Pharmacogenomics, Department of Molecular Neuropharmacology, Maj Institute of Pharmacology Polish Academy of Sciences, Kraków, Poland; 3 Laboratory of Pharmacogenomics and Individualized Therapy, Department of Pharmacy, School of Health Sciences, University of Patras, Patras, Greece; 4 Department of Pharmaceutical Biotechnology, School of Pharmacy, Guilan University of Medical Sciences, Rasht, Iran; 5 Department of Medical Genetics and Molecular Medicine, School of Medicine, Mashhad University of Medical Sciences, Mashhad, Iran; 6 Student Research Committee, Faculty of Medicine, Mashhad University of Medical Sciences, Mashhad, Iran; 7 Medical Genetics Research Center, Faculty of Medicine, Mashhad University of Medical Sciences, Mashhad, Iran; 8 Institute of Computer Science, University of Bialystok, Białystok, Poland; 9 Department of Population Medicine and Lifestyle Diseases Prevention, Medical University of Bialystok, Białystok, Poland; 10 Department of Cardiology, Medical University of Bialystok, Białystok, Poland; 11 Clinical Research Centre, Medical University of Bialystok, Białystok, Poland; 12 Zayed Center for Health Sciences, United Arab Emirates University, Al-Ain, United Arab Emirates; 13 Department of Genetics and Genomics, College of Medicine and Health Sciences, United Arab Emirates University, Al-Ain, United Arab Emirates

**Keywords:** Pharmacogenomics, High throughput DNA sequencing, Pharmacovariants, Functional assessment, Deep computational analysis, Artificial intelligence, Machine learning

## Abstract

**Background:**

This pilot study aims to identify and functionally assess pharmacovariants in whole exome sequencing data. While detection of known variants has benefited from pharmacogenomic-dedicated bioinformatics tools before, in this paper we have tested novel deep computational analysis in addition to artificial intelligence as possible approaches for functional analysis of unknown markers within less studied drug-related genes.

**Methods:**

Pharmacovariants from 1800 drug-related genes from 100 WES data files underwent (a) deep computational analysis by eight bioinformatic algorithms (overall containing 23 tools) and (b) random forest (RF) classifier as the machine learning (ML) approach separately. ML model efficiency was calculated by internal and external cross-validation during recursive feature elimination. Protein modelling was also performed for predicted highly damaging variants with lower frequencies. Genotype–phenotype correlations were implemented for top selected variants in terms of highest possibility of being damaging.

**Results:**

Five deleterious pharmacovariants in the *RYR1*, *POLG*, *ANXA11*, *CCNH*, and *CDH23* genes identified in step (a) and subsequent analysis displayed high impact on drug-related phenotypes. Also, the utilization of recursive feature elimination achieved a subset of 175 malfunction pharmacovariants in 135 drug-related genes that were used by the RF model with fivefold internal cross-validation, resulting in an area under the curve of 0.9736842 with an average accuracy of 0.9818 (95% CI: 0.89, 0.99) on predicting whether a carrying individuals will develop adverse drug reactions or not. However, the external cross-validation of the same model indicated a possible false positive result when dealing with a low number of observations, as only 60 important variants in 49 genes were displayed, giving an AUC of 0.5384848 with an average accuracy of 0.9512 (95% CI: 0.83, 0.99).

**Conclusion:**

While there are some technologies for functionally assess not-interpreted pharmacovariants, there is still an essential need for the development of tools, methods, and algorithms which are able to provide a functional prediction for every single pharmacovariant in both large-scale datasets and small cohorts. Our approaches may bring new insights for choosing the right computational assessment algorithms out of high throughput DNA sequencing data from small cohorts to be used for personalized drug therapy implementation.

**Supplementary Information:**

The online version contains supplementary material available at 10.1186/s40246-023-00508-1.

## Introduction

The genomics revolution, caused by the advancement of high throughput sequencing technologies, resulted in unravelling several novel genetic variants in pharmacogenomics (PGx) studies [1, 2]. While clinical, evidence-based reports are still the gold standard for assigning a true functional outcome to most drug-related variants, computational assessments for functional interpretation of a vast number of pharmacovariants (genetic variants in drug-related genes), obtained through advanced genotyping methods, are truly considered by many investigators and research groups as the main approach in the field [3–5]. Since the number of identified variants in pharmacodynamic (PD), pharmacokinetic (PK), or drug absorption, distribution, metabolism, and excretion (ADME) genes is rapidly increasing, the necessity for integration of computational genomics into clinical PGx tests will be needed more than before [6]. However, two main barriers in this area still need to be addressed: (a) common bioinformatics tools, like SIFT, Polyphen2, Provean, CAAD, Mutation Assessor, etc., are not suitable for functional evaluation of every pharmacovariant and doing subsequent haplotype/diplotype calling and phenotype prediction [7]. (b) PGx dedicated software and algorithms like Stargazer, Aldy, PharmCAT, etc., are limited to particular genes and specific numbers of known variants [8–10]. Recent studies have reported the utilization of multi-tools and artificial intelligence approaches that may help in decoding potential malfunction alleles in drug-related genes [11, 12]. Applying deep learning (which is the utilization of a neural network in a collection of machine learning algorithms) has been proposed for the prediction of personalized treatment outcomes and drug-dosage modification as well [13]. Nevertheless, computational prediction of drug response is heavily dependent on the available data from the patients.

The current study employs the utilization of multiple bioinformatics tools and random forest machine learning [14] approaches on 100 whole exome sequencing (WES) data files, along with clinical information from cardiovascular disease patients and a healthy control cohort for unravelling novel PGx markers of adverse drug reactions (ADRs) in less studied, drug-related genes. The two approaches were used separately for analysis of variants identified in just one patient and/or repeated in several patients. Our workflow may help other researchers, who investigate “not very well-known” PD, PK, or ADME genes to design a method for classifying large-scale genotyping data and finding malfunction alleles in a fast and easy way. We also introduced “Gene Walking” as a novel, helpful approach for predicting pathogenic/likely pathogenic effect(s) of new and unreported and/or not functionally annotated variants within drug-related genes.

## Methods

### Data collection

Exome sequencing results from our previous study on comprehensive clinical PGx profiling of a cohort of 100 individuals, comprised of 50 cardiovascular disease patients with pulmonary hypertension and ischemic diseases, using a particular list of drugs (with/without ADRs), and 50 healthy people, were used in the current investigation [15]. Our study has been approved by the Bioethics Committee of the Medical University of Białystok (approval number R-I-002/630/2018). Demographic information for all participants and data concerning clinical manifestation for patients with ADRs were obtained. Known and actionable SNPs were decoded by PGx-dedicated bioinformatic algorithms and reported previously [15]. The rest of the genomic markers (unknown/not interpreted within PGx area) are used in the current manuscript for unravelling potential impactful variants in drug-related genes.

### Data filtration

A type of custom filtered VCF files were used in the current study. The related setting for filtration of VCF files described below. Based on previous reports on the limitations of common bioinformatics tools to identify and highlight different types of altered pharmacovariants (especially for those which are responsible for intermediate and ultrarapid metabolizers), after some initial assessment, we did an extensive pre-filtration on the original WES VCF files for 1800 drug-related genes in the human genome. The genes within the list were collected from the PharmGKB [16] comprehensive gene list (only genes with at least one annotated variant extracted), (*n* = 1707), CPIC gene-drug records (*n* = 119), and the table of “Pharmacogenomic Biomarkers” from FDA for drug labelling (*n* = 132). Also, a systematic search within PubMed for possible newly introduced but not completely annotated/interpreted as an evidence-based record was performed while preparing our comprehensive drug-related gene list. We used the keywords: “Pharmacogenomics genes, Pharmacogenetic gene, drug-related gene, drug metabolizer gene, drug transporter gene, drug target gene, personalized medicine + gene, personalized therapy + gene, individualized therapy + gene” for studies published after 2021. The abstracts were screened to check if the selected keyword expansion were related to PGx context. Finally, full-text article assessed for the genes of direct implication on PGx research. After combining input from all sources, duplicate genes were removed. Next, the related BED file including the genes’ symbols along with the related genomic coordinates and positions in a “.CSV” format was created by the BioMart tool in ENSEMBL 105. Finally, the BCFtools V.1.15.1 package [17] used for massive filtration of VCF files for drug-related genes only. The outcome is named PGx-VCFs which contained only the variants in genes, related to drug metabolism, transferring, targeting, and receptors. PGx-VCFs then used in both common bioinformatic tools (see the next section for the names) and machine learning steps for functional assessment and identification of malfunction alleles (mostly loss of function, InDels, and short duplications).

**Deep computational analysis for extremely rare variants:**

#### In silico functional assessments

VEP [18] and SnpEFF [19] were initially applied on raw VCF files of WES, containing ~ 32,000 variants for each sample. Damaging variants were identified and compared to pathogenic/likely pathogenic variants in filtered VCF files later. VarSeq of Golden-Helix® [20] was utilized for molecular profiling of filtered VCFs (~ 3500 variants for each sample) through the following conditions:—rare variants were selected based on minor allele frequency (MAF) < 0.01 in 1 K Genomes [21], gnomAD (V.3.1.2), and ExAC (LOF) [22]—Heterozygotes and Homozygotes were separated and the read depth < 10 was assigned as low quality—Genotype quality ≥ 10 remained for further analysis—SIFT, Polyphen2, Mutation Taster, Mutation Assessor, FATHMM, and Provean scores, through the integration of dbNSFP 154v2 [23, 24], were applied for functional assessment of selected variants (see “Applying multiple bioinformatics tools” section for further details)—ExAC functional gene constraints 0.3, ClinVAR [25] haplotypes/variants 2021, and PharmGKB drug associations with the 2019 level of evidence were also included in the filtration steps.—CAAD 1.5 [26, 27] as an independent tool was applied for the filtered variants from the previous steps. The outcome considered novel damaging variants only from drug-related genes in our samples.

#### Additional data collection and gene walking

Filtered genes by VarSeq with finalized variants were used in BioMart again and the related BED file was employed for filtration of publicly available VCF files from 1 K Genomes, GET-RM [28], Complete-Genomics, Genome in a bottle consortium [29], KAVIAR [30], gnomAD, and ENSEMBL. A list of clinically associated markers was also obtained from PharmGKB and evaluated along with other VCF files for finding VarSeq introduced variants and their neighbour variants. We called this process "Gene Walking," as it follows the procedure of finding the nearest interpreted functional variant in the closest genomic coordinates to infer possible similar activity for an unknown target genomic marker. STRINGdb [31] and KEGG [32, 33] databases were also utilized for looking for genes functionally connected to our selected genes within cellular pathways.

#### Applying multiple bioinformatics tools

Next, a deep computational functional assessment of all selected variants within our selected genes was performed by free source annotation tools as well as Variant Validator [34], VarSome [35], ENSEMBL variant table, ACMG [36], ClinVar, gnomAD, and OMIM clinical features. The tools applied in following sequence: Ensembl variant table, gnomAD, Variant Validator, Varsome, ACMG classifier as part of VarSome, ClinVar, and OMIM. Different settings for each of these bioinformatic tools were as follow as well: for the ENSEMBL, we tracked the variant within “variant table” for the related genes. Then, “deleterious” assigned to the variant if 4-6/6 annotation tools (SIFT, PolyPhen, CADD, REVEL, MetaLR, and Mutation Assessor) predicted that as pathogen/damaging. The SIFT score ranges from 0.0 (deleterious) to 1.0 (tolerated). Variants with scores in the range between 0.0 and 0.05 are considered deleterious. The PolyPhen, on the other side, assigns the scores within ranges from 0.0 (tolerated) to 1.0 (deleterious) but variants with scores of 0.0 are predicted to be benign. Values closer to 1.0 are more confidently predicted to be deleterious. CADD provides score ranges from 1 to 99, higher values indicating more deleterious outcome. Scores above 30 are considered deleterious. The REVEL score for an individual variant can range from zero to one; missense variants with a REVEL score above 0.5 are considered damaging while missense variants with a REVEL score below 0.5 are considered tolerated. The MetaLR score can range from 0 to 1, when higher values are more likely to be deleterious. Missense variants with scores > 0.5 are classified as deleterious and missense variants with scores < 0.5 are classified as benign. Mutation Assessor score range is between 0 and 1 and variants with higher scores (closer to 1) are more likely to be deleterious. gnomAD v3.1.2 (GRCh38) dug for the selected variants and both “Variant Effect Predictor” and “In Silico Predictors” including SIFT, PolyPhen, REVEL, CADD, SpliceAI, and PrimateAI taken into account for determination of deleterious effect of variant. AI tools assign the score from 0.0 to 1.0, while closer to 1.0 is deleterious. “Population Frequencies” tables also checked for confirming the low allele frequency of evaluated variants. Genomic coordinate plus altered allele used as input variant description in validator tool from Variant Validator. After confirmation of related data, links to external resources for OMIM and VarSome obtained and followed, respectively. Through Varsome, we checked the “Germline Variant Classification” and entered the pathogenic, likely pathogenic and uncertain significant variants into the list. Again, both the pathogenicity scores and frequencies of exomes and genomes assessed and deleterious variants selected. To end of this point, the OMIM clinical features for each variant in addition to ClinVar categorization on pathogenic or uncertain significance for them were added to our list. Duplicates were removed from the result of different tools’ interpretation. Then, a list of pathogenic/likely pathogenic variants with the highest damaging scores from chosen genes were prepared out of previous step and assigned as the input for the VarAFT tool [37] (containing Annovar, CADD, SIFT, PolyPhen2, Mutation Taster, Mutation Assessor, Eigen, FATHMM, GERP++, LRT, PROVEAN, SiPhy, UMD-prediction, VEST3, and ClinSIG score). For “Variant Type” in VarAFT, exonic, splicing, synonymous, nonsynonymous, stoploss, stopgain, frameshift deletion, frameshift insertion, and frameshift sub selected from Refseq model. For the “Frequency” within the public databases, we included gnomAD E-All- < = 0.01 and 1000G- < = 0.01. In “Prediction” category, all the damaging and deleterious plus unknown options selected. CADD > = 15, DANN > = 0.9, Eigen > = 1, and GERP++ > = 2 assigned by default and other tools as well as SIFT, PolyPhen, UMD predictor, Mutation Taster, etc., set for damaging, pathogenic or probable pathogenic. Also, “Human Splice Finder” only included probable effect and most probable effect on splicing options. “Genes Information” followed the setting of RVIS score < = 0.25, LoFTool < = 0.01, GHIS > = 0.5, and GDI score low for all disease. As expected, amino acid substitution might result in protein misfolding, instability, trafficking, aberrant protein–protein interactions and affect protein’s function negatively. The result of VarAFT underwent protein modelling for proving negative effects for variants from selected genes in our patients as well.

#### Control samples

A set of 39 well-known pharmacovariants (validated in PharmVAR 5.1 [38]) in 11 very important pharmacogenes (VIPs) comprised the control group. PGx markers in *CYP2B6*, *CYP2C19*, *CYP2C9*, *CYP2D6*, *CYP3A5*, *F5L*, *SLCO1B1*, *DPYD*, *TPMT*, *UGT1A1*, and *VKORC1* were selected and examined by bioinformatics tools used in the previous steps to check the capability of such algorithms to reveal actionable and/or annotated pharmacovariants.

#### Applying homology modelling

In this stage, we first modelled protein to visualize the main conformational effect of amino acid substitution. Additionally, we analysed the effect of variants on hydrogen bonds (H-bonds) to adjacent residues using a Swiss pdb viewer (version 4.1.0) and evaluate possible changes in functional outcome of amino acid substitution. A total of five missense variants, the most highly pathogenic (received highest scores of damaging by bioinformatic tools) and phenotype-related in our patients, were modelled via SWISS-MODEL tools [39] using the best appropriate templates, chosen according to the results of the reference protein blast using NCBI BLAST (BLASTP 2.13.0+). Next, the designed models were visualized by Pymol1.1 software [40]. The mutated and wild type proteins were modelled and compared to demonstrate negative effects of altered amino acids on protein structures.

#### Haplotype/diplotype identification

The linkage disequilibrium (LD) calculator of Ensembl was used for displaying the LD results among the variants of interest from the deep computational analysis steps. According to the Ensembl variation resources, the calculated LD results are based on the 1000 Genomes Project.

### Machine learning for PGx variants

#### Input data and machine training

The PGx-VCF files were also mined and transformed into a meaningful table subsequently used to train the predictive algorithm for the classification of genomic variants in drug-related genes that may act as the potential pharmacovariants for developing ADRs in carriers. The initial dataset had 23,615 variants (variables). However, before machine training, all the VCFs underwent an extra three step filtration consisting of: removing non-informative variants (variables identified only in 1, 2 ,3 or all patients), a chi-squared test between the group (ADRs or not) and the existence or not of a variant genotype, and recursive feature elimination (RFE) to further decrease the number of variables and select only statistically meaningful markers. The outcome was employed as the training set and was assessed with fivefold internal cross-validation in the random forest (RF) model. Similar simulated external data were used for external cross-validation (fivefold cross-validation with 10 iterations) to check the reliability and significance of our developed model.

#### PGx phenotype prediction

ClinVar, OMIM, and Phenolayzer [41] were considered for identification of any association with phenotype data (ADRs in our patients). Drug-drug conflicts and gene-drug interactions for final interpreted phenotypes or PGx alleles in all participants were also assessed, using registered demographic data and complete history of intake medicines plus clinical manifestations in the case of patients with reported ADR phenotypes. Different sources including: DRUGBANK [42], PharmGKB, Flockhart table [43], and Drugs.com were employed for such measurements in details. SIDER 4.1 of EMBL [44] was also utilized for listing possible or existing side-effects for drugs used by our patients.

## Results

### Multiple bioinformatics tools outcome

**Fig. 1:**
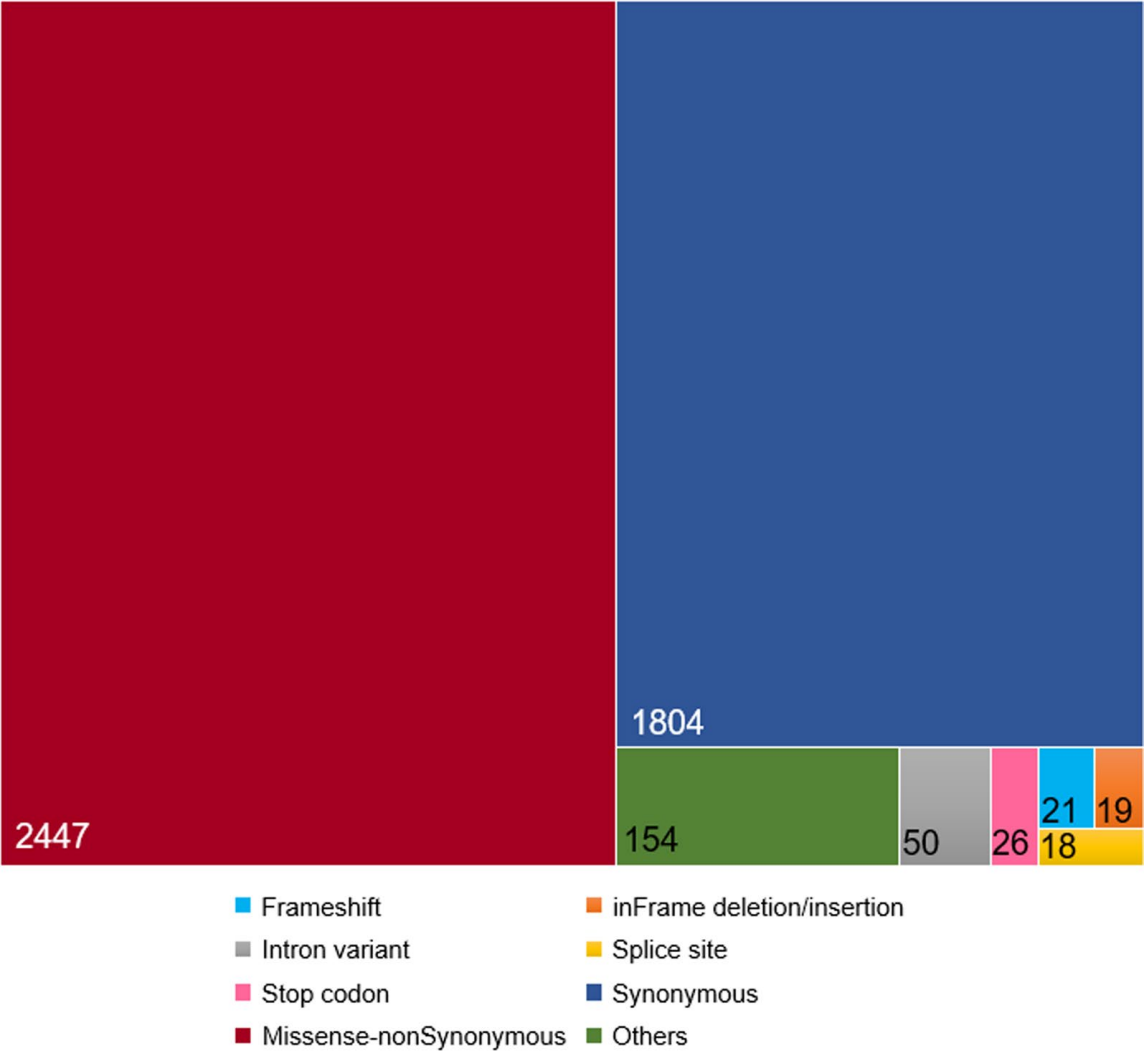
Rare variants in WES data. Frequency and functional impact of identified rare pharmacovariants within PGx-VCFs (see the text for further information). WES: whole exome sequencing, PGx: pharmacogenomics

**Fig. 2:**
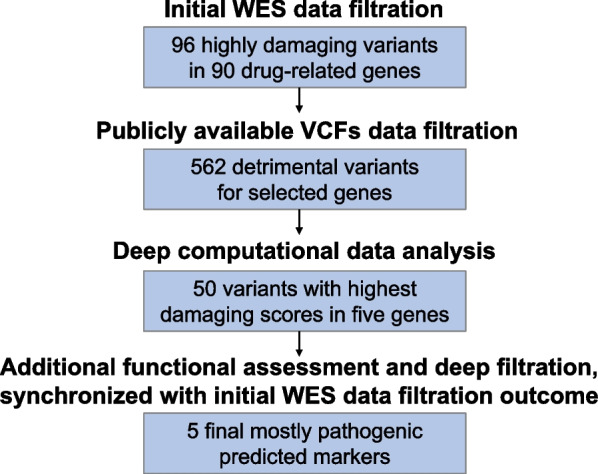
WES data deep filtration and computational functional assessments. Exome sequencing data were initially filtered for 1800 drug-related genes and analysed by VarSeq, including multiple functional prediction tools as well as SIFT, PolyPhen2, Mutation Assessor, Mutation Taster, FATHMM, and CAAD. Next, several publicly available VCF files were collected and filtered for VarSeq selected genes by related BED file. Frequency of variants in the VarSeq result was assessed in public VCFs and data for neighbour markers were gathered as well. Deep computational data analysis was performed by 23 bioinformatics tools and algorithms for all variants from the previous step. Final data analysis and filtration were performed in order to extract the five most pathogenic markers within the genes with damaging variants in patients with ADRs. (See the text for further information). WES: whole exome sequencing, ADR: adverse drug reaction

The control study for the software revealed the ability of common bioinformatics tools to identify loss of function pharmacovariants more than other types of PGx markers in selected genes. According to this fact, the designed algorithm in VarSeq for PGx-VCFs detected 96 highly damaging variants in 90 less-studied drug-related genes within the participants’ samples (the list of genes and related variants’ genomic coordinates are available in Additional file 1). Figure 1 illustrates the distribution and functional impact of all rare variants (including the highlighted 96) in filtered VCFs, which contain only drug-related genes. Also, extraction of all 96 selected variants and previously interpreted neighbour markers for each, obtained from public VCF files, resulted in the identification of 562 detrimental variants (gene walking outcome). Deep computational functional assessments (in silico assessment) of all 562 variants then revealed 351 pathogenic/likely pathogenic, 54 benign/likely benign, and 106 variants of unknown significance, plus 51 unreported variants before duplications were removed. Finally, a list of the top 50 variants with the highest damaging scores in five genes with consistent data for selected ADRs was regained and 5 final most pathogenic predicted markers were isolated after re-analysing the list of VarAFT top 50 variants (rs2230641, rs201076440, rs1049550, rs113994096, and rs775643457). Figure 2 also displays the outcome of each computational analysis step in our workflow in detail.

#### Protein modelling and structural analysis

**Fig. 3:**
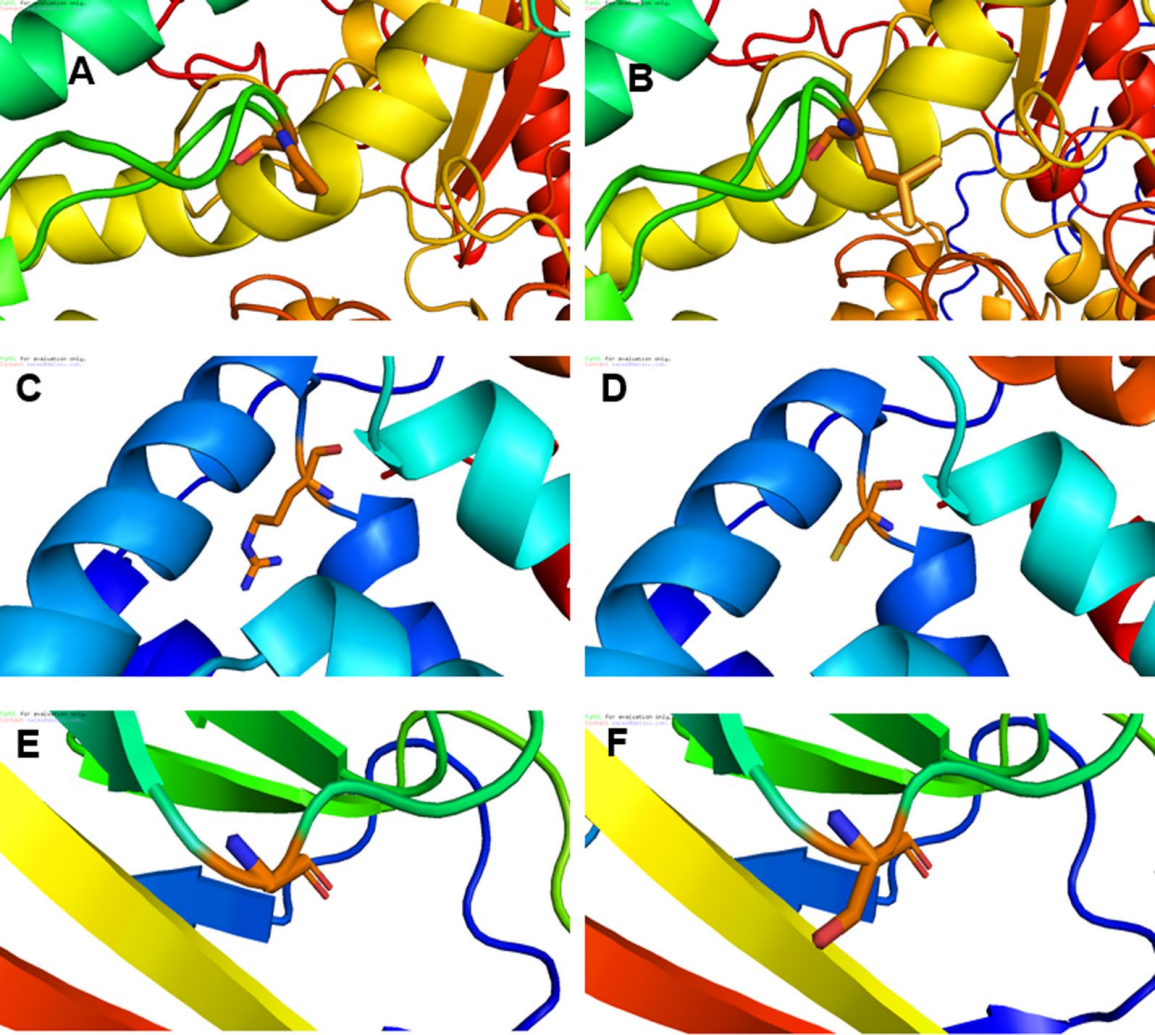
Homology modelling for three selected pharmacovariants in deep computational analysis. Close view of three damaging variants with potential influence on changing protein structure and functions in selected genes: **A** Wild type POLG protein, produced using 3IKM as the template, close view of Pro587. **B** Mutated POLG (p. Pro587Leu) protein model, produced using 3IKM as the template. **C** Wild type annexin A11 protein modelled using 6TU2 as the template, close view of Arg230. **D** Mutated annexin A11 protein (p. Arg230Cys) modelled using 6TU2 as the template. **E** Wild type EC 26 domain of cadherin-23 protein modelled using mouse cadherin-23 structure (5WJM) as the template, close view of Gly2771. **F** Mutated EC 26 domain of cadherin-23 protein (p. Gly2771Ser), modelled using mouse cadherin-23 structure (5WJM) as the template

The predicted outcomes of five variants on protein structure alteration were explored as follows: *RYR1*:p.Arg1954His, modelled using rabbit RYR1 (PDB ID = 5GKY) as the template [45], demonstrated the basic arginine alternation to basic histidine. *POLG*:p.Pro587Leu modelling showed substitution of proline, which is an evolutionary conserved residue [46] and nonpolar amino acid, to leucine, a nonpolar and branched amino acid in the linker domain. The modelling of *ANXA11*:p.Arg230Cys illustrated basic arginine substitution to nonpolar cysteine in the annexin A11 annexin repeat domain, which is also highly conserved [47]. *CCNH*:p.Val270Ala leads to nonpolar Val270 alternation to a smaller nonpolar amino acid, Alanine. The EC 26 domain of the cadherin-23 protein was modelled to assess the *CDH23*:p.Gly2771Ser mutation as well, which showed that Glycine, a nonpolar amino acid, is altered to a polar residue, Serine. Also, analysis by Swiss-Pdb Viewer revealed that the H-bond length has changed in all of the mutated proteins except for POLG:p.P87L. The Pro87 residue does not create H-bonds to other residues, either in wild type or in mutated (p.P87L) form. In the ANXA11:p.R230C and CDH23:p.Gly2771Ser mutated protein, the H-bonds for Arg230-Ser229, and Gly2771-Glu2773 do not exist, respectively. In CCNH:p.Val270Aal, the length of one H-bond has increased (Ala270-Arg266) and while another one has decreased (Ala270-Lys274). Although in RYR1:p.R1954H the Arg1954-Gly2130 H-bond and some of Arg1954-Glu1950 H-bonds are disrupted, a new His1954-Val2070 H-bond is formed. Table 1 presents the homology modelling features in detail. Three alterations of *ANXA11*:p.Arg230Cys, *CCNH*:p.Val270Aal, and *CDH23*:p.Gly2771Ser resulted in changes in protein conformation as well. Hence, structural modifications and abnormal activities in drug processing may be expected for these variants. Figure 3 displays changes in POLG and ANXA11 proteins as a result of amino acid alterations in two conserved residues, in addition to a transformed amino acid in CDH23 as a polarity alteration.

**Table 1:** Details of homology modelling for five detected deleterious variants in drug-related genes

Gene	Transcript ID	Protein ID	Variant		Affected domain	Residues	H-Bonds length to adjacent residues					PDB ID	Template			Template references
			Systematic name	Alternative/protein name									Coverage (%)	Identity (%)	Resolution (Å)	
*RYR1*	NM_000540.3	NP_000531.2	c.5861G > A	p.Arg1954His	–	His1954 (p.R1954H)	Glu1950	Leu1951	Leu1958	Val2070	Gly2130	5GKY	97	96.54	3.80	[45]
							2.81 Å	3.29 Å	3.06 Å	3.28 Å	–					
							2.65 Å	3.30 Å	2.99 Å	–	2.88 Å					
							2.65 Å									
						Arg1954 (WT)	3.21 Å									
							2.58 Å									
*POLG*	NM_002693.3	NP_002684.1	c.1760C > T	p.Pro587Leu	linker domain	Leu87 (p.P87L) Pro87 (WT)	No H-Bond No H-Bond					3IKM	94	99.83	3.24	[48]
*ANXA11*	NM_145868.2	NP_665875.1	c.688C > T	p.Arg230Cys	Annexin repeat	Cys230 (p.R230C)	Arg235			Ser229		6TU2	62	96.51	2.30	–
							2.71 Å			–						
						Arg230 (WT)	2.71 Å			2.95 Å						
*CCNH*	NM_001239.4	NP_001230.1	c.809 T > C	p.Val270Ala	C-terminal	Ala270 (p.Val270Aal)	Lys274			Arg266		1KXU	100	100	2.6	[49]
							2.91 Å			3.01 Å						
						Val270 (WT)	2.81 Å			3.06 Å						
*CDH23*	NM_022124.6	NP_071407.4	c.8311G > A	p.Gly2771Ser	EC 26 (Extracellular cadherin)	Ser2771 (p.Gly2771Ser) Gly2771 (WT)	Glu2773 – 3.07 Å					5WJM	39 (of domain)	41.5	2.9	[50]

#### Haplotype and linkage disequilibrium for selected variants

Upon testing the variants rs2230641, rs201076440, rs1049550, rs113994096, and rs775643457 with the linkage disequilibrium calculator of Ensembl, it was noticed that two variants (more specifically, rs201076440 and rs775643457) had no 1000 Genomes data. Referring to the variant rs1049550 and the variant rs2230641, the linkage disequilibrium calculator gave r2 = 0.110263 and *D*' = 0.604979. This is considered to be a low D' and r2, so no strong LD is predicted among them. That is mostly caused from different chromosome locations. The rs1049550 and rs2230641 are missense variants, and their location within the human genome is 10:80166946 and 5:87399457, respectively.

#### Machine learning outcome

The limitation of the sample size used as the training set (175 pharmacovariants in 135 genes within 50 patients with ADRs) could not be ignored as the RFE method was utilized for variable selection. At first, extra filtration in three steps resulted in reducing the initial variants to 9861 (informative variants), 187 (statistically significantly different between the two sets of patients), and 175 (from the RFE process), respectively. The latter were the final variants list indicated by the RF model. This subset of variants achieved an average accuracy of 0.9818 (95% CI: 0.84, 0.98—area under the curve (AUC) 0.9736842, area under the precision-recall curve (prAUC) 0) when predicting whether a patient would develop any ADRs or not with the following metrics:

**Table:** 

Variables	Accuracy	Kappa	AccuracySD	KappaSD
175	0.9818182	0.95849057	0.04065578	0.09281792

However, the utilization of a similar simulated dataset in the form of external cross-validation in the designed model in the next step resulted in the introduction of only 60 variants in 49 genes as the important markers with potential effects on drug metabolism pathways. This outcome was highlighted with the lower AUC of 0.5384848 with accuracy of 0.9512:

**Table:** 

Variables	Accuracy	Kappa	AccuracySD	KappaSD
60	0.9512195	0.8918206	0.2987183	0.0627351

**Fig. 4:**
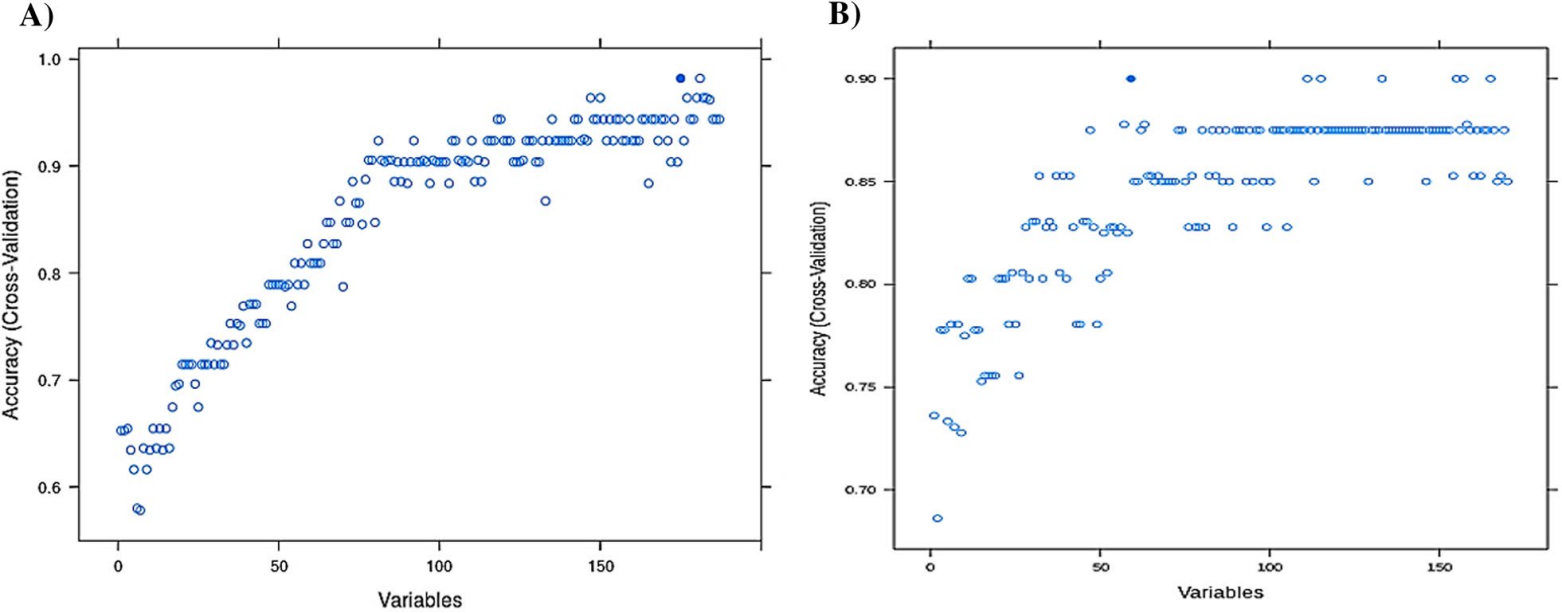
The comparison of average accuracy for the designed ML model by means of both internal and external cross-validation. Accuracy of the prediction model for developing ADRs in cardiovascular patients demonstrated by different cross-validation approaches. **A** The RFE process of machine learning reduced the variables to 175 important genotype variants. These are the final variants indicated by the RF model, which employed internal fivefold cross-validation. **B** The accuracy changed during the testing of the designed model by external cross-validation and the number of important pharmacovariants reduced to 60. The subset of the variants achieved an average accuracy of 0.9818 and 0.9512 on predicting whether a patient will have ADRs or not, respectively. ML: machine learning, ADR: adverse drug reactions, RFE: recursive feature elimination, RF: random forest

**Fig. 5:**
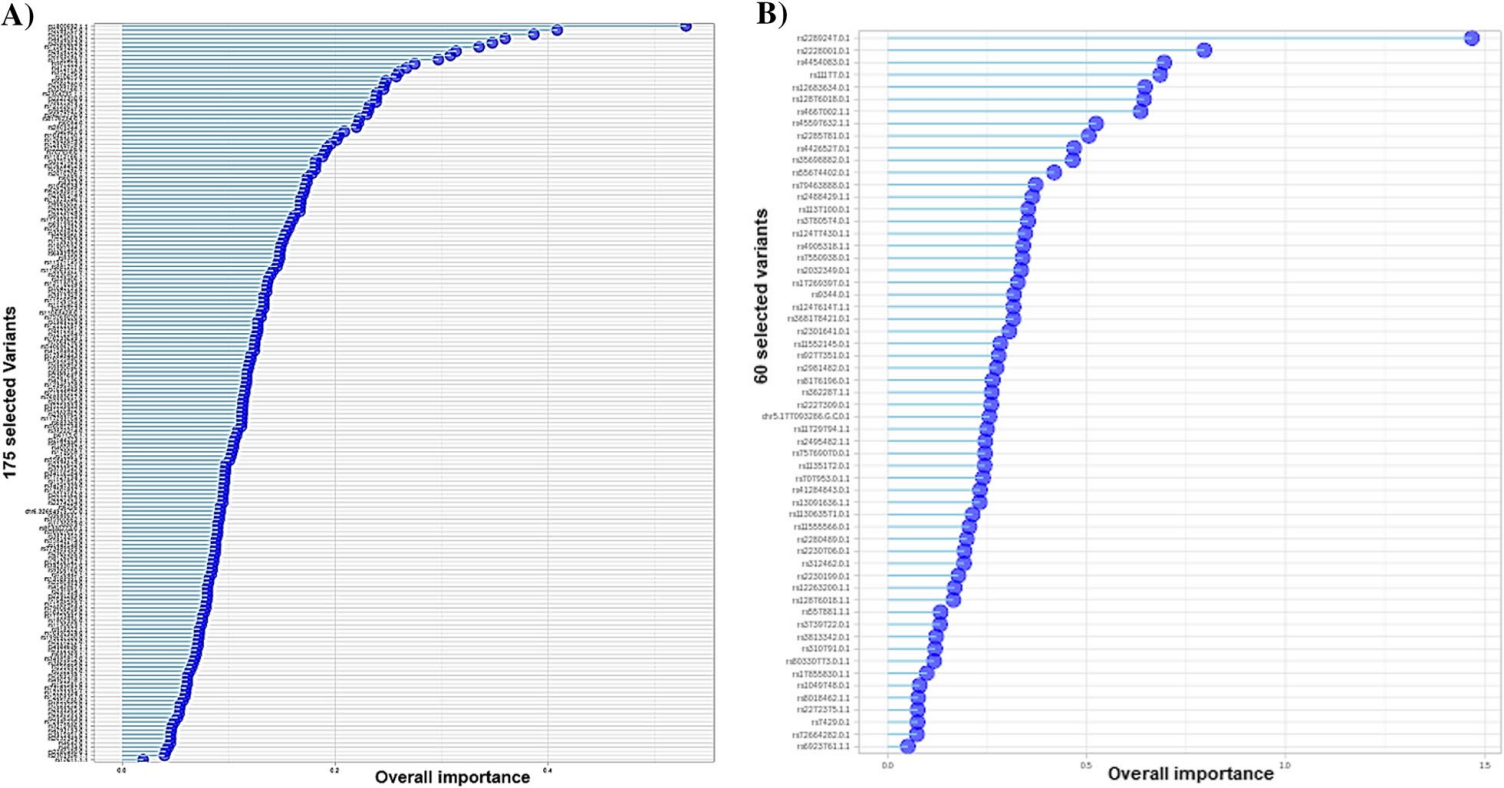
Comparison of ML final selected variants’ importance. 175 final variants by internal and 60 variants by external cross-validation, introduced by the RF model as the important variables that may cause ADRs in cardiovascular patients who received particular drugs. The differences and low accuracy for external validation must be considered while applying machine learning for small cohorts (see the text for the discussion). Associated importance values are available in Additional file 3. ML: machine learning, ADR: adverse drug reactions, RF: random forest

Figure 4 demonstrates the comparison of average accuracy for the designed model by means of both internal and external cross-validation. The compared statistics from confusion matrixes of the final deployed model are shown in Table 2 and the importance of each variant genotype variable for the model is visualized in Fig. 5.

**Table 2:** The statistics of confusion matrixes of the final deployed RF model for both internal and external cross-validation

	Internal cross-validation	External cross-validation
Accuracy	0.9808	0.9512
95% CI	(0.8974, 0.9995)	(0.8347, 0.994)
No information rate	0.6346	0.6341
P-value (ACC > INR)	1.664e-09	2.309e-06
Kappa	0.9581	0.8918
McNemar’s test P-value	1.0000	0.4795
Sensitivity	1.0000	0.8667
Specificity	0.9474	1.0000
Pos pred value	0.9706	1.0000
Neg pred value	1.0000	0.9286
Precision	0.9705882	1.0000000
Recall	1.0000000	0.8666667
F1	0.9850746	0.9285714
Prevalence	0.6346	0.3659
Detection rate	0.6346	0.3171
Detection prevalence	0.6538	0.3171
Balanced accuracy	0.9737	0.9333
Area under the curve (AUC)	0.9736842	0.5384848

Note that while the accuracies in both types of validation are quite high, the overfitting to the training data within internal cross-validation resulted in an unreal AUC. On the other hand, increasing the sample size with external cross-validation displayed more “close to real” performance of the RF model for small cohorts

*‘Positive’ Class* Patients with ADRs, *AUC* area under the curve, *RF* random forest

#### Genotype–phenotype correlation/predictions

A total number of 278 known genes were identified as drug related genes, based on all drugs used by the examined patients, as obtained from DRUGBANK and PharmGKB. The evaluation of drug-drug interactions by Drugs.com and the Flockhart table, plus common side-effects by SIDER 4.1, revealed no negative effects for the specific drugs and resembling observed ADRs in related patients. The genes linked to the drugs were synced to our comprehensive 1800 gene list in order to check if they were found using our two different approaches as well. However, since the final selected genes and related pharmacovariants in our results were not part of so-called actionable PGx genes, allele imputation and genotype–phenotype correlation could not benefit from reference databases: *CPIC*, *DPWG*, and *PharmVAR*. Therefore, the results of evaluations performed by ClinVar, OMIM, and Phenolayzer were considered for running genotype–phenotype correlations and make predictions. Some adverse effects in patients were reported as linked clinical manifestations to the variants in our selected genes. Additional file 2 displays the data used for making genotype–phenotype correlations, along with additional information concerning drug history for ADR patients in our study.

## Discussion

Several rare genetic variants within drug-related genes are anticipated to play important roles in variability in drug responses among individuals. Detection of such genetic biomarkers is continuously increasing through the utilization of NGS technologies in the clinic [6]. Innovative technologies for data mining and computational genomic characterizations have paved the way for understanding the relationship between the human genome and drug-related phenotype. The ability of computational approaches in drug repurposing for some specific medications has been demonstrated before [51]. The current investigation also, by confirming the utilization of multi-bioinformatics tools, may aid in the discovery of novel and rare pharmacovariants in NGS-derived data and in providing the link between genetic background and clinical manifestations for both rare and common PGx markers within drug-related genes. However, the clinical value and utility of such approaches must be evaluated before heading toward implementation in healthcare systems.

In silico tools have been proven a useful platform for large-scale genomic data mining and addressing the identification of functional similarities between various genes and variants [52], classifying and assessment of potential pathogenicity for novel and not interpreted variants, functional characterization of incidental findings (IFs) and variants of unknown significance (VUS) in different populations with the highest levels of genetic diversity [53]. Yet, not all genomic markers (especially pharmacovariants) are located within evolutionary conserved genomic coordinates, and thus would not provide straight input data for bioinformatic analysis. Because of that, we applied an adapted methodology for related PGx data pre-filtration and employed numerous computational algorithms, including the innovative approach of gene walking described herein, with the aim of focusing on recognizable genomic markers and their functionality assessments in extremely rare variants within pharmacogenes. Although gene walking is not expected to demonstrate one hundred percent true functional consequences of novel variants, it may get us closer to the potential cellular activity of each variant, especially when the two markers are located within the same coding part of the gene.

Also, 3D modelling for structurally altered proteins may add additional insights about damaging effects of pharmacovariants on related molecules. Changing in residues with crucial role in protein conformation, polarity, stability, and function will result in negative outcomes in handling the related substrates (drugs) within cells. The clinical manifestations and patients’ phenotype confirm/support the predicted consequences as well [54]. In all of our five selected variants, amino acid substitution seems to affect protein stability, especially in the ANXA11:p.R230C, CDH23:p.Gly2771Ser, and RYR1:p.R1954H with altered H-bonds which may lead to changing in free energy levels (altered ΔΔG value). However, no modification in H-bonds but altered evolutionary conserved residue (POLG structure with mutated variant) may still decrease the protein stability and pose a negative function for that [55].

It is also noteworthy that predictions made using machine learning approaches proved to be highly sensitive to the input data used for training the algorithm. Moreover, to illustrate the true clinical utility of a technology, clinical randomized trials that compare treatment outcomes through the utilization of artificial intelligence-derived therapy versus traditional approaches or guideline-based treatment must be applied [56]. Random forest methods for in silico assessment of pathogenicity for more complex variants in not evolutionary conserved genes (as well as drug-related genes) have been proposed before [57]. Our data, however, demonstrated the potential disadvantages of such approaches in rare pharmacovariant detection and classification when there are a low number of observations (patients with/without ADRs). Even though there was an initial desired result for the model, low reliability of such approaches in small cohorts must be taken into account, especially since it is necessary to run external cross-validation to check the significance of the model, whether or not complete phenotypic data for the patients exists.

Registration of patients’ PGx data in local electronic healthcare records (EHR) has already been achieved and the PGx card developed as a novel digitalization system for quick access to such data [58–60]. The current study also added detailed information on novel and/or not previously interpreted variants in less studied drug-related genes into a newly designed local database for participants’ PGx actionable data [15]. Included data are as follows: applied genotyping technology and bioinformatics tools, genomic position and frequency for the variants, pathogenicity classification, variant consequences, genome-built assembly, and the output of functional assessment based on American College of Medical Genetics and Genomics/the Association for Clinical Genomic Science (ACMG/ACGS) guidelines. Also, considerations were added (for research use only) for possible interactions and conflicts with current treatment outcomes plus “links to update” data on the related gene in PharmGKB. An example of anonymous data is available within https://www.clinicalpgx.pl/data.

Although comprehensive DNA sequencing technologies like WGS or WES can lead to more in-depth exploration of genomic and pharmacogenomic data, some intrinsic complications like IFs and VUS still pose problems for the results. The current investigation also revealed several completely novel variants with no available primary annotation at all. Further processing of such potential biomarkers was not possible as our approaches initially relied on already existing information for partial assessment to continue our analysis. Moreover, machine learning only focuses on variants with more frequencies within our samples and simply ignores those variants that are seen only in one sample. Furthermore, statistical reports are thoroughly affected by the number of observations, resulting in false positive outcomes and overfitted models. This is expected to be seen when there is a large number of features compared to the sample size. Although different steps were applied to the data for reducing the number of variables and dimensional reduction of features in the current study, common practice in statistics rely on at least 100 observations to do the related statistical analysis (i.e., in RF models) and significance accuracy to be attained. Indeed, finding and collecting patients with ADRs and registered clinical manifestations would be another challenge in terms of time and multicentre collaboration. In addition, complexity and heterogeneity of the data causes discrepancies between internal and external cross-validation AUC values as well. Even though the first computational method was adapted for analysing variants neglected by ML, which may be the causative markers for carrying individuals, the statistical analysis may also be affected by sample numbers in different ways. Specially, when some false negative results for PGx markers appear while using common bioinformatic algorithms for detection.

In addition, haplotype analysis may yield no result using available LD calculator tools. This will happen when in silico approaches bring a few variants of interest in separate genomic coordinates with no evidence of any correlation between them. Although the beginning of the era of large-scale genotype data and experimental phasing has caused the identification of haplotypes and LD to be regarded with great importance, with possible applications in the field of clinical pharmacogenomics, it is still possible to detect no significant data in a narrow area of the likely candidate region of the human genome with functional variants of interest. Currently, PHASE, FastPHASE, BEAGLE, MATCH, and IMPUTE2 are among the statistical methods with a high impact in modelling population haplotype frequencies of unrelated individuals for computational phasing [61–63]. The more individuals taken into consideration, the better the final estimation. For related individuals, identity by descent (IBM) could be informative for filling gaps in determining the haplotype phase. The association between pairs of sites, or loci, is the main point of LD, but the large-scale data era is providing information for associations between large intervening chromosome regions named long-range linkage disequilibria (LRLD). Sved and coworkers completed an analysis based on HapMap phase 3 data [64]. They concluded that possible associations between blocks on different chromosomes for particular regions might be observed [65, 66].

It has been proposed that bringing multi-omics data into PGx studies may result in more invaluable information on different regulatory mechanisms and further facilitation for drug discovery, especially in cancer PGx [67, 68]. Large amounts of high-dimensional data alongside the machine learning approaches for biomedical computing will fuel future research on genotype–phenotype correlations in the area of precision medicine [69]. Such methods would be expected to increase our understanding of PGx markers’ true functions within cellular pathways and related clinical outcomes as well [70].

However, advanced non-in silico analysis of PGx results may still demonstrate closer to real consequences of PGx variants. Because of that, deep initial computational filtration of large-scale genomic data to achieve a reduced number of the most potentially damaging markers for in vitro functional characterizations seems reasonable. Today, genome editing and CRISPR modified cell cultures for pharmacovariants within ADME genes have been introduced and the future of such methods speculated as well [71, 72]. The same can be applied for top scored damaging variants in the current study. Here, we may choose the cell culture media from the related tissue, which shows the highest amount of gene expression for our candidate variant, and analyse the outcome of the CRISPR-guided genetic mutation on drug metabolism as well.

## Conclusions

The prediction of functional outcomes for every single identified pathogenic/likely pathogenic genetic variant on drug response within high throughput DNA sequencing results is the major challenge for fast development of PGx guidelines and subsequent test implementation in the daily clinical setting. While some progress in computational analysis of large genomic variants has already been made, there is still an essential need for the development of tools, methods, and algorithms that are able to provide functional assessments for all pharmacovariants in both large-scale datasets and small cohorts while performing haplotype/diplotype inference and phenotype estimation. This development is crucial for the true integration of advanced genome profiling technologies, especially NGS-guided treatment modifications, into daily clinical practice. Artificial intelligence methods may help in finding hidden algorithms and patterns within PGx data and perform the clinical classification of rare pharmacovariants as well. But such approaches are clearly dependent on the type of input data and the number of observations. Advanced technologies may someday enable us to investigate gene-drug interactions even before medications are released on the market and used in clinic.

## Supplementary Information

**Additional file 1**. VarSeq selected novel damaging variants’ position range and related gene names.

**Additional file 2**. Demographic information, history of disease and drug treatment, and clinical manifestations for the patients who developed adverse drug reactions in the current study.

**Additional file 3**. Associated important variants with each cross-validation approach in machine learning model.

## Abbreviations

ACGS: The Association for Clinical Genomic Science
ACMG: American College of Medical Genetics and Genomics
ADME: Absorption, distribution, metabolism, and excretion
ADRs: Adverse drug reactions
HER: Electronic health records
IBM: Identity by descent
IF: Incidental findings
LD: Linkage disequilibrium
LRLD: Long-range linkage disequilibria
MAF: Minor allele frequency
ML: Machine learning
PD: Pharmacodynamic
PGx: Pharmacogenomic
PK: Pharmacokinetic
prAUC: Area under the precision-recall curve
RF: Random forest
RFE: Recursive feature elimination
VUS: Variants with unknown significance
WES: Whole exome sequencing
