# Supplementary material for: Pharmacovariome scanning using whole pharmacogene resequencing coupled with deep computational analysis and machine learning for clinical pharmacogenomics
Source: Hum Genomics. 2023 Jul 14;17:62. doi: 10.1186/s40246-023-00508-1 (PMC10347842; doi:10.1186/s40246-023-00508-1)
Supplement: Supplementary file 1 — Additional file 1. VarSeq selected novel damaging variants’ position range and related gene names. [file 40246_2023_508_MOESM1_ESM.docx]

**VarSeq selected novel damaging variants’ position range and related gene names:**

| **Chr:Pos** | **Gene Names** |
| --- | --- |
| 1:171199350-60  1:176670970-80  10:80162010-20  10:129759310-20  1:202963650-60  15:98913260-70  3:20126110-20  17:63489060-70  17:50684860-70  5:87363470-80  7:1898350-60  5:76195080-90  6:77463030-40  1:237784250-60  16:11060920-30  16:87838710-20  15:93000520-30  1:165680510-20  3:57429700-10  7:158799060-70  5:53042150-60  11:99956860-70  11:103220720-30  7:151035580-90  15:33785740-50  16:13947730-40  5:102261910-20  14:52314790-99  11:20103260-70  22:43928880-90  15:89314610-20  19:38505060-70  2:208239080-90  12:96022240-50  10:71807510-20  11:103220720-30  20:53254050-60  14:50915910-20  11:100299380-90  12:109444430-40  1:147201170-80  11:60089810-20  8:61548190-99  22:43928880-90  15:33827280-90  1:11144670-80  3:52802510-20  8:69832010-20  1:94001990-99  3:49358040-50  2:25240360-70  20:54157500-10  3:49358040-50  10:60196610-20  19:38543840-50  1:109261070-80  3:52802510-20  19:7172330-40  17:58272830-40  1:241860580-90  9:6588610-20  10:97030810-20  15:33785740-50  3:125110280-90  10:80162010-20  14:64210020-30  7:5997410-20  9:133647980-90  22:42884390-99  11:21534490-99  8:69638740-50  9:135778440-50  22:43928880-90  15:89325630-40  2:191039290-99  20:8647930-40  2:26693130-40  2:26693140-50  10:77834030-40  10:94268970-80  11:68386490-99  19:38561300-10  10:52315030-40  22:24513480-90  3:21425650-60  10:66069480-90  15:78533480-90  1:171199350-60  8:112408330-40  17:27760140-50  19:38543550-60  10:52315030-40  11:32396260-70  16:55505420-30  22:43928880-90  1:3412660-70  11:61795500-10  6:77463030-40  17:19555990-99  20:46009970-80  4:153705040-50  5:53051510-20  13:59992490-99  19:38490120-30  16:55669610-20  7:71677210-20  7:117627530-40  10:80162010-20  14:52314790-99 | *FMO2*  *PAPPA2*  *ANXA11*  *MGMT*  *CYB5R1*  *IGF1R*  *KAT2B*  *ACE*  *ABCC3*  *CCNH, RASA1*  *MAD1L1*  *SV2C*  *HTR1B*  *RYR2*  *CLEC16A*  *SLC7A5*  *CHD2*  *ALDH9A1*  *DNAH12*  *ESYT2*  *ITGA2*  *CNTN5*  *DYNC2H1*  *ABCB8*  *RYR3*  *ERCC4*  *SLCO4C1*  *PTGER2*  *NAV2*  *PNPLA3*  *FANCI*  *RYR1*  *IDH1*  *LTA4H*  *CDH23*  *DYNC2H1*  *TSHZ2*  *PYGL*  *CNTN5*  *MYO1H*  *CHD1L, FMO5*  *MS4A2*  *ASPH*  *PNPLA3*  *RYR3*  *MTOR*  *ITIH3*  *SLCO5A1*  *ABCA4*  *GPX1*  *DNMT3A*  *CYP24A1*  *GPX1*  *ANK3*  *RYR1*  *CELSR2*  *ITIH3*  *INSR*  *MPO*  *EXO1*  *GLDC*  *SLIT1*  *RYR3*  *SLC12A8*  *ANXA11*  *SYNE2*  *PMS2*  *DBH*  *PACSIN2*  *NELL1*  *SULF1*  *KCNT1*  *PNPLA3*  *POLG*  *STAT4*  *PLCB1*  *KCNK3*  *KCNK3*  *DLG5*  *PLCE1*  *LRP5*  *RYR1*  *DKK1*  *UPB1*  *ZNF385D*  *CTNNA3*  *HYKK*  *FMO2*  *CSMD3*  *NOS2*  *RYR1*  *DKK1*  *WT1*  *MMP2*  *PNPLA3*  *PRDM16*  *FEN1*  *HTR1B*  *SLC47A1*  *MMP9*  *TLR2*  *ITGA2*  *DIAPH3*  *RYR1*  *SLC6A2*  *GALNT17*  *CFTR*  *ANXA11*  *PTGER2* |
