# Supplementary material for: Pharmacovariome scanning using whole pharmacogene resequencing coupled with deep computational analysis and machine learning for clinical pharmacogenomics
Source: Hum Genomics. 2023 Jul 14;17:62. doi: 10.1186/s40246-023-00508-1 (PMC10347842; doi:10.1186/s40246-023-00508-1)
Supplement: Supplementary file 2 — Additional file 2. Demographic information, history of disease and drug treatment, and clinical manifestations for the patients who developed adverse drug reactions in the current study. [file 40246_2023_508_MOESM2_ESM.docx]

**Table 3:** Demographic information, history of disease and drug treatment, and clinical manifestations for the patients who developed adverse drug reactions in the current study.

| Patient number | Gender | Age | Height (cm) | Weight (Kg) | History of Cardiovascular Diseases | Intake Medicine(s) | Clinical Manifestation(s) after/during Treatment  (ADRs) |
| --- | --- | --- | --- | --- | --- | --- | --- |
|  |  |  |  |  |  |  |  |
| 1 | **2** | **79** | **168.4** | **74.2** | **Hypertension MI Peripheral arterial occlusive disease and intermittent claudication Angina (angina) / coronary heart disease / angina Varicose veins of the lower extremities Haemorrhagic diathesis** | **Aspirin Atorvastatin** | **Dizziness** |
| 2 | **2** | **78** | **153.6** | **64.7** | **Hypertension Elevated cholesterol Elevated triglyceride AF** | **Aspirin Allopurinol** | **Headache (5/10),**  **Diarrhoea (once),**  **Dizziness** |
| 3 | **1** | **43** | **173.8** | **124.9** | **Hypertension Elevated cholesterol Elevated triglyceride Vein thrombosis of the lower extremities** | **Aspirin** | **Coughing (once),**  **Headache (8/10),**  **Diarrhoea (once)** |
| 4 | **2** | **61** | **150.1** | **80.8** | **Hypertension Ventricular arrhythmia Varicose veins of the lower extremities Vein thrombosis of the lower extremities** | **Bisoprolol** | **Coughing (once),**  **Leg muscle cramp and ache** |
| 5 | **2** | **57** | **157.7** | **70.5** | **Hypertension** | **Ramipril** | **Dizziness** |
| 6 | **2** | **64** | **161.4** | **66.9** | **Elevated cholesterol Elevated triglyceride** | **Aspirin** | **Dizziness,**  **Stomach ache (5/10)** |
| 7 | **2** | **75** | **150.5** | **72.7** | **Hypertension Elevated cholesterol Elevated triglyceride Ventricular arrhythmia Varicose veins of the lower extremities** | **Aspirin** | **Headache (6/10),**  **Dizziness** |
| 8 | **1** | **77** | **176.9** | **84.3** | **Hypertension Elevated cholesterol Elevated triglyceride MI AF Ventricular arrhythmia Angina (angina) / coronary heart disease / angina Varicose veins of the lower extremities** | **Clopidogrel** | **Dizziness** |
| 9 | **2** | **62** | **161.4** | **80** | **Elevated cholesterol Elevated triglyceride** | **Bisoprolol** | **Leg muscle cramp and ache** |
| 10 | **2** | **74** | **162.7** | **83.3** | **Hypertension Elevated cholesterol Elevated triglyceride** | **Amlodipine** | **Coughing (once),**  **Dizziness** |
| 11 | **2** | **70** | **154.1** | **91.8** | **Hypertension Varicose veins of the lower extremities** | **Allopurinol** | **Dizziness** |
| 12 | **2** | **64** | **164.2** | **85** | **Varicose veins of the lower extremities** | **Aspirin** | **Nausea (once) Food withdrawal (once) Coughing, vomiting, and diarrhoea(twice),  Dizziness  Leg muscle cramp and ache** |
| 13 | **2** | **65** | **156** | **57.6** | **Elevated cholesterol** | **Rosuvastatin** | **Nausea (twice) Food withdrawal (twice) Vomiting and diarrhoea(twice) Coughing (once)** |
| 14 | **2** | **61** | **155.6** | **74.9** | **Hypertension Elevated cholesterol** | **Ramipril** | **Dizziness** |
| 15 | **2** | **72** | **147.5** | **85.8** | **Hypertension Elevated cholesterol MI Heart failure** | **Aspirin Rosuvastatin** | **Nausea (once) Food withdrawal (twice) Coughing, vomiting, and diarrhoea(twice),  Dizziness  Leg muscle cramp and ache Headache (5/10)** |
| 16 | **2** | **77** | **152** | **73.4** | **Hypertension Heart failure Varicose veins of the lower extremities** | **Bisoprolol** | **Nausea (twice) Food withdrawal (twice) Coughing and vomiting (twice) Diarrhoea (once)** |
| 17 | **1** | **38** | **181.4** | **88.9** | **Hypertension Elevated cholesterol** | **Ramipril** | **Nausea (twice) Food withdrawal (twice) Vomiting (twice) Diarrhoea (once) Coughing (once)** |
| 18 | **2** | **56** | **155.2** | **98.7** | **Hypertension** | **Ramipril** | **Diarrhoea (once) Coughing (once)** |
| 19 | **2** | **69** | **159.1** | **58.1** | **Hypertension Elevated cholesterol Elevated triglyceride Angina (angina) / coronary heart disease / angina Varicose veins of the lower extremities** | **Amlodipine** | **Dizziness  Coughing (once)** |
| 20 | **1** | **54** | **176.7** | **92.5** | **Stroke** | **Aspirin Bisoprolol Rosuvastatin** | **Headache (2/10) Leg muscle cramp and ache Coughing (once) Diarrhoea (once)** |
| 21 | **2** | **71** | **163.3** | **83.9** | **Hypertension** | **Bisoprolol** | **Leg muscle cramp and ache Coughing (once)** |
| 22 | **2** | **68** | **154.8** | **93.2** | **Hypertension Elevated cholesterol Elevated triglyceride Varicose veins of the lower extremities** | **Aspirin Bisoprolol** | **Headache (7/10) Leg muscle cramp and ache Coughing (once) Diarrhoea (once) Food withdrawal (once)** |
| 23 | **2** | **68** | **159.4** | **91.3** | **Hypertension Elevated cholesterol** | **Ramipril** | **Nausea (twice) Food withdrawal (twice) Vomiting (twice) Coughing (once) Diarrhoea (twice)** |
| 24 | **2** | **59** | **160** | **59.3** | **Hypertension Varicose veins of the lower extremities** | **Amlodipine Bisoprolol** | **Headache (10/10)** |
| 25 | **2** | **61** | **161.2** | **62** | **Elevated cholesterol Elevated triglyceride** | **Atorvastatin** | **Nausea (twice) Food withdrawal (once) Vomiting and diarrhoea(twice) Coughing (once) Dizziness** |
| 26 | **2** | **73** | **163.9** | **73.4** | **Hypertension Elevated cholesterol Elevated triglyceride Varicose veins of the lower extremities** | **Aspirin** | **Nausea (twice) Food withdrawal (twice) Vomiting (twice) Coughing (twice) Diarrhoea (once) Dizziness** |
| 27 | **2** | **61** | **164.4** | **68.4** | **Hypertension** | **Ramipril** | **Nausea (twice) Food withdrawal (once) Vomiting (twice) Coughing (twice) Diarrhoea (once)** |
| 28 | **1** | **70** | **179** | **85.9** | **?** | **Aspirin** | **Leg muscle cramp and ache Nausea (twice) Food withdrawal (twice) Vomiting (twice) Coughing (once) Diarrhoea (twice)** |
| 29 | **2** | **73** | **155.1** | **77.4** | **Hypertension Elevated cholesterol Elevated triglyceride** | **Aspirin** | **Leg muscle cramp and ache Nausea (twice) Food withdrawal (twice) Vomiting (twice) Coughing (twice) Diarrhoea (twice)** |
| 30 | **2** | **66** | **153.8** | **54.5** | **Hypertension Elevated cholesterol Elevated triglyceride Angina (angina) / coronary heart disease / angina Varicose veins of the lower extremities** | **Aspirin Rosuvastatin** | **Leg muscle cramp and ache Nausea (twice) Food withdrawal (twice) Vomiting (twice) Coughing (twice) Diarrhoea (twice)** |
| 31 | **1** | **55** | **169** | **91.1** | **Hypertension Elevated cholesterol** | **Rosuvastatin** | **Leg muscle cramp and ache** |
| 32 | **2** | **65** | **172.4** | **87.6** | **?** | **Bisoprolol** | **Nausea (twice) Food withdrawal (twice) Vomiting (twice) Coughing (twice) Diarrhoea (once)** |
| 33 | **1** | **48** | **178** | **85.6** | **Hypertension** | **Ramipril Allopurinol** | **Coughing (twice)** |

*- ADR: adverse drug reaction, Gender: 1=male & 2=female, MI: myocardial infarction, AF: Atrial fibrillation.*

*- Numbers in the clinical manifestations’ column indicate the severity of observed features in patients.*
